# Boosting Reaction Kinetics in Co_3_O_4_/ZnCo_2_O_4_ Frameworks with Heterostructures for High-Performance Lithium-Ion Batteries

**DOI:** 10.3390/ma19143148

**Published:** 2026-07-22

**Authors:** Qibei Tu, Zhifeng Wang

**Affiliations:** “The Belt and Road Initiative” Advanced Materials International Joint Research Center of Hebei Province, School of Materials Science and Engineering, Hebei University of Technology, Tianjin 300401, China; 202321801084@stu.hebut.edu.cn

**Keywords:** lithium-ion batteries, transition metal oxides, heterostructure, anode

## Abstract

When metal oxides are employed as anodes in lithium-ion batteries, their practical application is often constrained by sluggish reaction kinetics. Structure optimization and heterointerface regulation are effective strategies for improving the aforementioned issue. Herein, a series of Co_3_O_4_/ZnCo_2_O_4_ heterostructured materials with hollow structures is prepared. The effects of the two-phase ratio on the interfacial activity and electrochemical performance are systematically investigated. Among them, the optimized Co_3_O_4_/ZnCo_2_O_4_-2 material exhibits enhanced interfacial interactions and abundant oxygen vacancies, which optimize the local electronic environment and facilitate charge transfer. Electrochemical test results indicate that the Co_3_O_4_/ZnCo_2_O_4_-2 anode maintains a reversible capacity of 582.4 mAh g^−1^ after 1000 cycles at 1 A g^−1^, demonstrating good cycling stability. Furthermore, the full cell assembled with a LiFePO_4_ cathode maintains a discharge capacity of 115.9 mAh g^−1^ after 100 cycles at 0.2 C, validating the practical application potential of the material. This work reveals the key role of interface regulation in boosting Li^+^ diffusion kinetics of transition metal oxides, providing new insights for the rational design of heterostructured anodes.

## 1. Introduction

The rapid development of portable electronic devices and electric vehicles has led to an increasing demand for lithium-ion batteries (LIBs) with high energy density and long cycle life [1,2]. Therefore, developing novel anodes with higher specific capacities has become a key focus of current research [3,4,5,6]. Among various candidate materials, Co_3_O_4_ has been recognized as a promising candidate for next-generation anodes in LIBs due to its high theoretical specific capacity (890 mAh g^−1^), abundant resources, and environmental friendliness [7]. However, the severe volume expansion during charge/discharge processes causes pulverization of the electrode structure and active material detachment, resulting in significant capacity decay [8]. On the other hand, its inherent semiconductor characteristics result in low electronic conductivity and sluggish lithium-ion diffusion kinetics, severely limiting its rate capability. Therefore, addressing these two issues simultaneously is crucial for achieving high-performance applications of Co_3_O_4_ anodes [9,10,11].

The construction of hollow structures has proven to be a highly effective strategy. Hollow structures provide ample internal buffer space, effectively accommodating volume changes during cycling and thereby maintaining the structural integrity of the electrode. Zhu et al. designed Co_3_O_4_ hollow nanoparticles immobilized on N,S-codoped reduced graphene oxide sheets (HoCo_3_O_4_/NS-RGO) [12]. This material maintained a specific capacity of 1590 mAh g^−1^ after 600 cycles over 1 A g^−1^, confirming that the hollow structure helps to shorten the Li^+^ transport pathway within the electrode. Li et al. designed a hollow Co-Co_3_O_4_ wrapped with a CNT layer, which can mitigate the volume changes of Co_3_O_4_ and boost its conductive performance [13]. Although structural optimization alone can improve cycling stability, its enhancement of electronic conductivity remains limited. Accordingly, further modification approaches are needed to address these limitations.

To improve diffusion kinetics, the construction of heterostructures has become a research hotspot in recent years [14,15]. Heterointerfaces can induce interfacial charge redistribution and form built-in electric fields, thereby accelerating electron/ion transport. Liu et al. synthesized Co_3_O_4_/Fe_3_O_4_ composites with coherent heterointerfaces [16]. The Co_3_O_4_/Fe_3_O_4_ heterostructure electrode could accelerate Li^+^ diffusion and significantly improve transport kinetics. Zhang et al. synthesized in situ hybrid NiO/Co_3_O_4_ porous nanoflowers via a thermal treatment method [17]. The two components exhibited a synergistic effect, with NiO providing a stable flower-like framework and Co_3_O_4_ reducing charge transfer resistance. This material demonstrated superior lithium storage performance compared to single-component anodes. Moreover, bimetallic oxides not only provide abundant electrochemically active sites due to the introduction of a second metal cation but also further enhance lithium storage capability through synergistic effects [18,19]. Chen et al. developed a simple solvothermal method to in situ synthesize hollow Co_3_O_4_/NiCo_2_O_4_ heterostructured flowers [20]. Based on band theory, the semiconductor oxide heterostructure formed by Co_3_O_4_ and NiCo_2_O_4_ can enhance electronic conductivity and improve Li^+^ transport efficiency. However, previous studies mainly focus on composites with a single phase ratio. Similar studies on heterostructures composed of bimetallic oxides and the control of their composition ratios remain insufficient.

In this work, hollow Co_3_O_4_/ZnCo_2_O_4_ heterostructure materials were successfully constructed by precisely controlling the molar ratio of Zn/Co. The effects of the two-phase ratio on the structure, interfacial activity, and electrochemical performance were systematically investigated. It was found that when the two-phase ratio was optimized, the material (denoted as Co_3_O_4_/ZnCo_2_O_4_-2) presented exceptional electrochemical properties. DFT calculations further confirm the charge transfer promotion at the heterointerface. The Co_3_O_4_/ZnCo_2_O_4_-2 retains a reversible capacity of 863.4 mAh g^−1^ after 200 cycles at 0.2 A g^−1^. When assembled into a full cell, it also exhibits excellent cycle stability. This work constructs a Co_3_O_4_/ZnCo_2_O_4_ heterostructured anode through hollow structure engineering and heterointerface design. The synergy between interfacial charge redistribution and oxygen vacancies is found to enhance Li^+^ diffusion kinetics, providing deeper insight into interface engineering of bimetallic oxide anodes.

## 2. Experimental

### Synthesis Method

**Figure 1:**
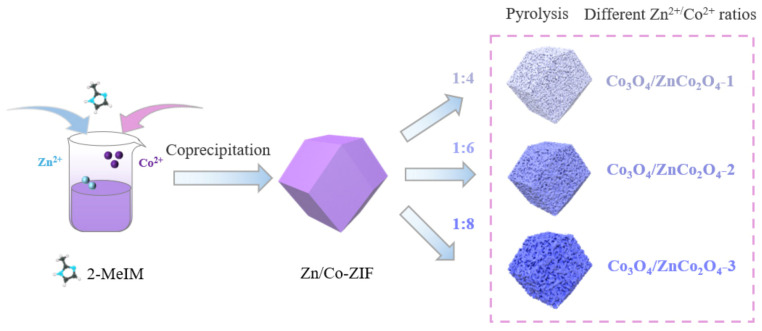
Schematic for the synthesis of Co_3_O_4_/ZnCo_2_O_4_ with different ratios.

The synthesis steps are illustrated in Figure 1. First, according to a Zn^2+^ to Co^2+^ molar ratio of 1:6, 0.169 g of Zn(NO_3_)_2_·6H_2_O and 0.991 g of Co(NO_3_)_2_·6H_2_O were dissolved in 200 mL of methanol (denoted as solution A), while 1.300 g of 2-methylimidazole was dissolved in 80 mL of methanol (denoted as solution B). Solutions A and B were quickly mixed and stirred for 24 h. After reaction, the precipitate was collected and washed with methanol. Finally, the product was dried for 12 h to obtain the Zn/Co-ZIF precursor. Moreover, precursor solutions with Zn^2+^ to Co^2+^ ratios of 1:4 and 1:8 were also prepared under the same conditions.

The as-obtained Zn/Co-ZIF powder was subjected to thermal treatment in air atmosphere. The temperature was ramped to 350 °C at 2 °C min^−1^ and held for 2 h to obtain the final products. The resulting products derived from Zn/Co-ZIF precursors with Zn^2+^ to Co^2+^ molar ratios of 1:4, 1:6, and 1:8 are denoted as Co_3_O_4_/ZnCo_2_O_4_-1, Co_3_O_4_/ZnCo_2_O_4_-2, and Co_3_O_4_/ZnCo_2_O_4_-3, respectively. The amount of Zn^2+^ gradually decreases, leading to a corresponding decrease in the relative content of the ZnCo_2_O_4_ phase in the final products. Further details on material characterization, electrochemical measurements, and DFT calculations can be found in the Supporting S1 [21,22,23,24,25].

## 3. Results and Discussion

**Figure 2:**
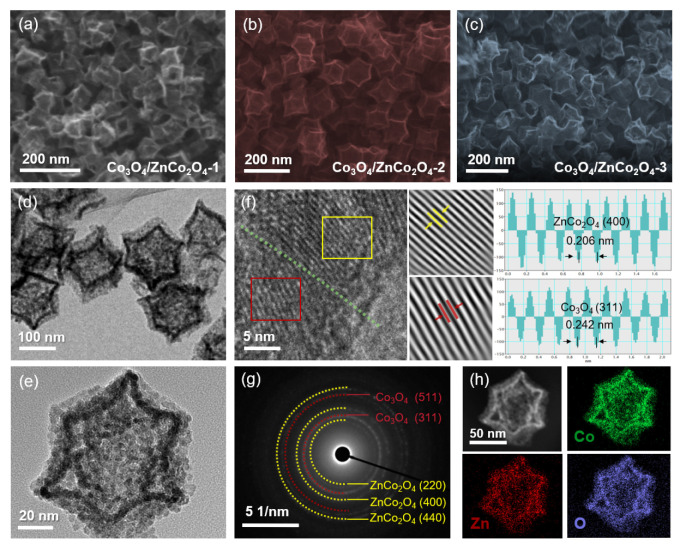
SEM images of (**a**) Co_3_O_4_/ZnCo_2_O_4_-1; (**b**) Co_3_O_4_/ZnCo_2_O_4_-2 and (**c**) Co_3_O_4_/ZnCo_2_O_4_-3. (**d**,**e**) TEM images of Co_3_O_4_/ZnCo_2_O_4_-2. (**f**) HRTEM image of Co_3_O_4_/ZnCo_2_O_4_-2. (**g**) SAED pattern and (**h**) elemental mapping of Co_3_O_4_/ZnCo_2_O_4_-2.

SEM was employed to examine the morphology of the as-prepared samples. Figure S1 shows the SEM image of the Zn/Co-ZIF precursor (Zn/Co = 1:6), which exhibits a well-defined dodecahedral morphology with a smooth surface, exhibiting a mean size of around 100 nm. Figure 2a–c present the SEM images of Co_3_O_4_/ZnCo_2_O_4_ with different two-phase ratios. All three samples generally retain the polyhedral morphology, though partial breakage and deformation are observed in Co_3_O_4_/ZnCo_2_O_4_-1 and Co_3_O_4_/ZnCo_2_O_4_-3. In contrast, Co_3_O_4_/ZnCo_2_O_4_-2 exhibits the most intact morphology, suggesting that an appropriate two-phase ratio favors structural integrity, which can help alleviate structural collapse and ensure stable electrochemical performance during cycling. The elemental compositions of three Co_3_O_4_/ZnCo_2_O_4_ samples were examined by EDS and ICP analyses (Tables S1 and S2). Based on these results, the relative mass fractions of Co_3_O_4_ and ZnCo_2_O_4_ were estimated, which are approximately 1:5.5, 1:1.6, and 1:1.2 for Co_3_O_4_/ZnCo_2_O_4_-1, Co_3_O_4_/ZnCo_2_O_4_-2, and Co_3_O_4_/ZnCo_2_O_4_-3, respectively.

TEM was used to further probe the microstructure of the Co_3_O_4_/ZnCo_2_O_4_-2. As displayed in Figure 2d,e, the Co_3_O_4_/ZnCo_2_O_4_-2 exhibits a hollow structure with a surface composed of stacked small nanoparticles, between which obvious voids can be observed. This morphology primarily arises from the decomposition of organic ligands during calcination [26]. This hollow porous structure is beneficial for alleviating volume changes during cycling and enlarging the electrode–electrolyte contact area, which boosts the electrochemical performance. Figure 2f presents the HRTEM image of Co_3_O_4_/ZnCo_2_O_4_-2. Two sets of lattice fringes with different orientations can be clearly observed. The measured interplanar spacings are 0.242 nm and 0.206 nm, corresponding to the (311) plane of Co_3_O_4_ and the (400) plane of ZnCo_2_O_4_, respectively. This result confirms the coexistence of Co_3_O_4_ and ZnCo_2_O_4_, thereby constructing a two-phase heterostructure. The heterointerface can facilitate a reduction in the interfacial charge transfer barrier. The SAED pattern in Figure 2g shows polycrystalline diffraction rings that can be assigned to specific crystal planes of Co_3_O_4_ and ZnCo_2_O_4_. Elemental mapping reveals the uniform dispersion of Zn, Co, and O (Figure 2h). Such a uniform distribution favors sufficient contact between the Co_3_O_4_ and ZnCo_2_O_4_ phases at the interface, which can enable the construction of abundant heterointerfaces.

**Figure 3:**
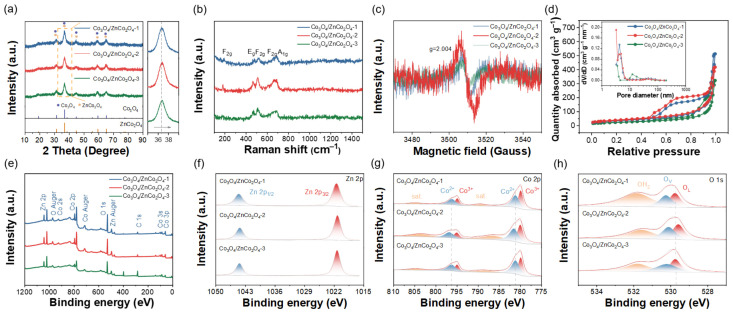
(**a**) XRD pattern, (**b**) Raman spectra and (**c**) EPR spectra of three samples. (**d**) N_2_ adsorption–desorption isotherms and pore size distribution (inset). (**e**) XPS survey and high-resolution XPS spectra of (**f**) Zn 2p; (**g**) Co 2p and (**h**) O 1s of three samples.

XRD analysis was conducted to determine the phase composition (Figure 3a). All observed diffraction peaks are well consistent with the standard patterns of Co_3_O_4_ (PDF#74–2120) and ZnCo_2_O_4_ (PDF#23–1390). The five diffraction peaks in the figure can be indexed to the (220), (311), (400), (511), and (440) crystal planes of both phases. No additional peaks corresponding to Zn-related impurities are detected, suggesting that Zn predominantly exists as ZnCo_2_O_4_. To further clarify the influence of different ratios on the crystal structure, slow scan XRD was performed in the 2θ range of 34–40° at 1° min^−1^. The results reveal a shift in the diffraction peaks toward higher 2θ angles with decreasing ZnCo_2_O_4_ ratio. This may arise from the larger ionic radius of Zn^2+^ relative to Co^2+^. The reduction in Zn^2+^ content leads to lattice contraction and decreased interplanar spacing, resulting in the observed peak shift [27]. Raman spectra are presented in Figure 3b. All samples display comparable Raman scattering signals, with characteristic peaks located at approximately 193, 477, 522, 613, and 688 cm^−1^. Both Co_3_O_4_ and ZnCo_2_O_4_ have a similar structure; their Raman spectral features are essentially consistent. Among these, the F_2g_ and A_1g_ modes are assigned to Co-O vibrations in tetrahedral and octahedral coordination, respectively, while the E_g_ mode is associated with the intense stretching modes of Co-O and Zn-O bonds [28,29].

EPR spectroscopy was performed to characterize the vacancy defects of the three samples (Figure 3c). Among them, Co_3_O_4_/ZnCo_2_O_4_-2 exhibits the strongest EPR signal, corresponding to abundant oxygen vacancies. The strongest EPR signal of Co_3_O_4_/ZnCo_2_O_4_-2 can be attributed to the substitution of Co^2+^ by Zn^2+^, which induces local lattice distortion. Additionally, the interaction at the two-phase interface promotes charge redistribution and electron accumulation, also facilitating the formation of oxygen vacancies [30]. The presence of oxygen vacancies plays a role in improving the electrical conductivity of the Co_3_O_4_/ZnCo_2_O_4_-2. N_2_ adsorption–desorption measurements reveal that all three materials display typical type III isotherms with distinct H3 hysteresis loops (Figure 3d), indicating a mesoporous structure. The pore size distribution curves (inset) show that Co_3_O_4_/ZnCo_2_O_4_-2 has an average pore size of approximately 8 nm. Moreover, it exhibits the highest specific surface area, reaching 145.4 m^2^ g^−1^, higher than those of Co_3_O_4_/ZnCo_2_O_4_-1 (127.5 m^2^ g^−1^) and Co_3_O_4_/ZnCo_2_O_4_-3 (85.6 m^2^ g^−1^). The larger specific surface area stems from the preserved polyhedral morphology and hollow structure, which facilitate sufficient electrolyte penetration and promote rapid Li^+^ transport, thereby contributing to enhanced cycling stability.

The elemental states of the three materials were further analyzed by XPS. The survey spectra show characteristic peaks corresponding to Zn, Co, and O, with no signals from other elements detected (Figure 3e). The high-resolution Zn 2p spectra exhibit peaks at 1021.6 eV and 1044.7 eV, corresponding to Zn 2p_3/2_ and Zn 2p_1/2_ of Zn^2+^, respectively (Figure 3f). The Co 2p high-resolution spectra display two main peaks at 780.2 eV and 795.1 eV, assigned to Co 2p_3/2_ and Co 2p_1/2_, respectively (Figure 3g). Moreover, the Co^2+^ peaks in Co_3_O_4_/ZnCo_2_O_4_-2 shift toward higher binding energy. This is due to the stronger Co-O bonding in ZnCo_2_O_4_ relative to that in Co_3_O_4_, leading to an intrinsic difference in the electronic environment of Co between the two phases. When the two phases are in contact, charge transfer occurs at the interface. Co_3_O_4_/ZnCo_2_O_4_-2 possesses an optimized two-phase ratio, which induces stronger electronic interactions at the interface and thus results in a more pronounced peak shift [31,32]. Three peaks are resolved in the O 1s spectra (Figure 3h): lattice oxygen at 529.6 eV (O_L_), adsorbed water oxygen at 531.8 eV (OH_2_), and oxygen vacancies at 530.1 eV (O_V_), which verifies the presence of oxygen vacancies. The shift of O_L_ toward lower binding energy further confirms the change in the electronic environment. Quantitative XPS analysis (Table S3) reveals that the Co^2+^/Co^3+^ ratio of the three samples follows the trend of ZnCo_2_O_4_ content. Additionally, Co_3_O_4_/ZnCo_2_O_4_-2 shows the highest O_V_/O_L_ ratio, indicating the highest surface oxygen vacancy concentration.

**Figure 4:**
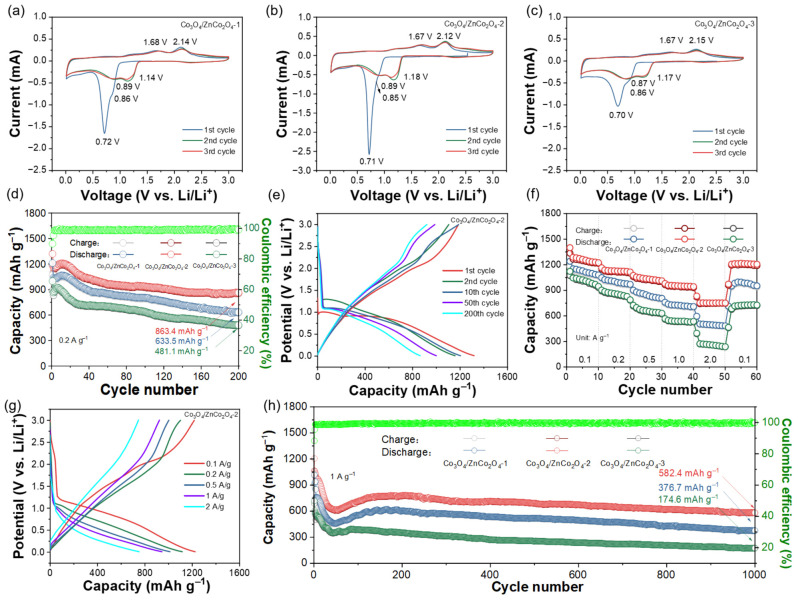
CV curves of (**a**) Co_3_O_4_/ZnCo_2_O_4_-1; (**b**) Co_3_O_4_/ZnCo_2_O_4_-2 and (**c**) Co_3_O_4_/ZnCo_2_O_4_-3. (**d**) Cycling performance of three samples at 0.2 A g^−1^. (**e**) The GCD curves of Co_3_O_4_/ZnCo_2_O_4_-2 for different cycles. (**f**) Rate performance. (**g**) The GCD curves of Co_3_O_4_/ZnCo_2_O_4_-2 at different current densities. (**h**) Long-term cycling performance at 1 A g^−1^ of three samples.

The influence of the two-phase ratio on electrochemical performance was assessed through CV tests in the voltage range of 0.01–3.0 V at 0.2 mV s^−1^ (Figure 4a–c). The CV curves of all three materials exhibit characteristic redox peaks of Co_3_O_4_ and ZnCo_2_O_4_, with similar peak shapes and positions. Taking the Co_3_O_4_/ZnCo_2_O_4_-2 anode as an example, two reduction peaks are observed during the first discharge process. The peak at 0.85 V is associated with the conversion reaction of Co_3_O_4_ to Co. The peak at 0.71 V corresponds to the irreversible reduction of ZnCo_2_O_4_ to Zn and Co, along with the alloying reaction between Zn and Li, accompanied by the buildup of the solid electrolyte interphase (SEI) film [33]. During the first charge process, two distinct oxidation peaks are observed. The peak near 1.67 V is associated with the oxidation of Co to Co^2+^ and the oxidation of Zn, while the peak at 2.12 V corresponds to the further oxidation of Co^2+^ to Co^3+^. Compared to the first scan, the reduction peaks of all three anodes shift to higher potentials with broader peak shapes in subsequent scans. This originates from electrochemical activation and the reduction in crystallite size during the initial lithiation process [34,35]. The CV curves nearly overlap after the second cycle, reflecting excellent reaction reversibility for all three materials. Moreover, Co_3_O_4_/ZnCo_2_O_4_-2 exhibits the strongest current response, which can be ascribed to its optimized two-phase ratio that promotes interfacial electronic interactions and thereby accelerates reaction kinetics. In contrast, Co_3_O_4_/ZnCo_2_O_4_-3 exhibits the weakest current response, likely due to its less favorable two-phase ratio that hinders interfacial interactions. The compromised structural integrity also weakens its charge transfer kinetics. The reaction equations are as follows:

(1) $${\mathrm{Co}}_{3}O_{4}\;+\;{8\mathrm{Li}}^{+}\;+\;{8e}^{-}\;↔\;3\mathrm{Co}\;+\;{4\mathrm{Li}}_{2}O$$

(2) $${\mathrm{ZnCo}}_{2}O_{4}+{8\mathrm{Li}}^{+}+{\;8e}^{-}\;\to \;\mathrm{Zn}+2\mathrm{Co}+{4\mathrm{Li}}_{2}O$$

(3) $$\mathrm{Zn}+{\mathrm{Li}}^{+}+e^{-}\;↔\;\mathrm{LiZn}$$

(4) $$\mathrm{Zn}+{\mathrm{Li}}_{2}O\;↔\;\mathrm{ZnO}+{2\mathrm{Li}}^{+}+{\;2e}^{-}$$

(5) $$2\mathrm{Co}+{\;2\mathrm{Li}}_{2}O\;↔\;2\mathrm{CoO}+{4\mathrm{Li}}^{+}+{4e}^{-}$$

(6) $$2\mathrm{CoO}+{2/3\mathrm{Li}}_{2}O\;↔\;2/3{\mathrm{Co}}_{3}O_{4}+{\;4/3\mathrm{Li}}^{+}+{\;4/3e}^{-}$$

Figure 4d presents the cycling behavior of the three anodes tested at 0.2 A g^−1^. Among them, the Co_3_O_4_/ZnCo_2_O_4_-2 anode exhibits the highest reversible capacity, with initial discharge and charge capacities of 1318.5 and 1185.8 mAh g^−1^, respectively. This enhanced reversible capacity can be attributed to the more abundant two-phase interfaces generated by interfacial regulation, which strengthen the heterointerface interactions and provide extra active sites, thereby enhancing lithium storage capability. In contrast, Co_3_O_4_/ZnCo_2_O_4_-3 shows the lowest reversible capacity, due to its lower Zn content, which limits the capacity contribution from the alloying reaction. During the initial cycles, the reversible capacities of all three anodes exhibit a gradual increasing trend, probably arising from progressive electrode activation and the generation of an electrochemically induced gel-like polymer layer that enhances lithium storage. As cycling proceeds, the capacities gradually decline, possibly due to the decomposition of the polymer layer. After 200 cycles, Co_3_O_4_/ZnCo_2_O_4_-2 maintains 863.4 mAh g^−1^, surpassing those of Co_3_O_4_/ZnCo_2_O_4_-1 (633.5 mAh g^−1^) and Co_3_O_4_/ZnCo_2_O_4_-3 (481.1 mAh g^−1^). Moreover, it delivers the highest areal capacity of 0.87 mAh cm^−2^ among the three anodes (Table S4). The superior stability of Co_3_O_4_/ZnCo_2_O_4_-2 stems from its intact hollow structure, which effectively mitigates volume changes during charge–discharge processes and preserves structural integrity. However, the particles of Co_3_O_4_/ZnCo_2_O_4_-1 and Co_3_O_4_/ZnCo_2_O_4_-3 suffer from damage and structural collapse upon cycling, resulting in significant capacity decay.

The GCD curves of the three anodes are presented in Figure 4e and Figure S2. The initial coulombic efficiency of the Co_3_O_4_/ZnCo_2_O_4_-2 anode is 89.9%. The partial capacity loss is associated with SEI film formation on the electrode and incomplete Li_2_O decomposition [36]. The relatively high coulombic efficiency can be attributed to the following factors. The hollow structure facilitates electrolyte penetration and accommodates volume changes, promoting complete conversion reactions. The optimized heterointerface facilitates charge transfer, while the abundant oxygen vacancies further enhance the reaction kinetics. These factors collectively reduce side reactions during the first cycle, leading to an improved initial coulombic efficiency. The charge–discharge curves become almost coincident after the second cycle, indicating that the structure becomes stable during cycling. In subsequent cycles, the charge–discharge plateaus of the Co_3_O_4_/ZnCo_2_O_4_-2 anode remain well preserved, confirming its excellent stability. In contrast, the curves of the Co_3_O_4_/ZnCo_2_O_4_-1 and Co_3_O_4_/ZnCo_2_O_4_-3 anodes show more pronounced decay with increasing cycle number, which reflects their poor structural stability.

To evaluate the reliability of the data, electrochemical tests were performed with three parallel cells. Figure S3a shows the discharge capacities of three batteries from the same batch for selected cycles at 0.2 A g^−1^, and the corresponding standard deviations are displayed in Figure S3b. The detailed data for additional batches are further summarized in Table S5. Although slight variations in capacities exist among different cells, their electrochemical performance remains stable, as reflected by the small standard deviation, confirming the good reproducibility of the electrochemical measurements.

The rate capability was further evaluated at various current densities (Figure 4f). The Co_3_O_4_/ZnCo_2_O_4_-2 delivered discharge capacities of 1287.9, 1164.7, 1075.6, 960.6, and 753.5 mAh g^−1^ at 0.1, 0.2, 0.5, 1, and 2 A g^−1^, respectively. When the current density was reduced back to 0.1 A g^−1^, the capacity recovered to 1207.7 mAh g^−1^, showing the good stability of Co_3_O_4_/ZnCo_2_O_4_-2 under high-rate testing. In contrast, the Co_3_O_4_/ZnCo_2_O_4_-1 and Co_3_O_4_/ZnCo_2_O_4_-3 anodes exhibited more severe capacity decay at the high current density. Figure 4g presents the charge/discharge profiles of the Co_3_O_4_/ZnCo_2_O_4_-2 anode at various current densities. It can be clearly observed that even at 2.0 A g^−1^, the curves maintain a relatively intact shape without obvious distortion. This indicates that the Co_3_O_4_/ZnCo_2_O_4_-2 electrode can sustain a stable electrochemical reaction pathway under various rates.

Figure 4h shows the long-term cycling stability among three anodes with varying phase ratios. Among them, the Co_3_O_4_/ZnCo_2_O_4_-2 anode exhibits the highest initial capacity (1206.0 mAh g^−1^). During the initial cycles, the reversible capacities of all three anodes decrease, followed by a gradual increase and stabilization, which is a common feature of conversion-type anodes upon repeated cycling. The initial capacity decay results from the development of the SEI layer and depletion of the electrolyte. As cycling proceeds, the anodes become gradually activated, generating more active sites, which leads to capacity recovery and stabilization [37,38]. Throughout subsequent cycles, the Co_3_O_4_/ZnCo_2_O_4_-2 anode consistently demonstrates superior cycling stability, retaining 582.4 mAh g^−1^ after 1000 cycles. However, the Co_3_O_4_/ZnCo_2_O_4_-1 and Co_3_O_4_/ZnCo_2_O_4_-3 anodes retain only 376.7 and 174.6 mAh g^−1^, respectively, over 1000 cycles. The excellent cycling durability of the Co_3_O_4_/ZnCo_2_O_4_-2 anode benefits from its unique structural design and interfacial characteristics. First, the intact hollow structure enhances the mechanical stability of Co_3_O_4_/ZnCo_2_O_4_-2, enabling it to better accommodate volume changes during cycling and thereby mitigating capacity decay during charge/discharge processes [39]. Second, its optimized two-phase ratio strengthens interfacial interactions and suppresses stress accumulation during the cycling process. In contrast, the Co_3_O_4_/ZnCo_2_O_4_-1 and Co_3_O_4_/ZnCo_2_O_4_-3 anodes suffer from incomplete morphologies, making them less capable of withstanding repeated volume changes during cycling. Additionally, they have weak interfacial interactions and limited charge transfer.

**Figure 5:**
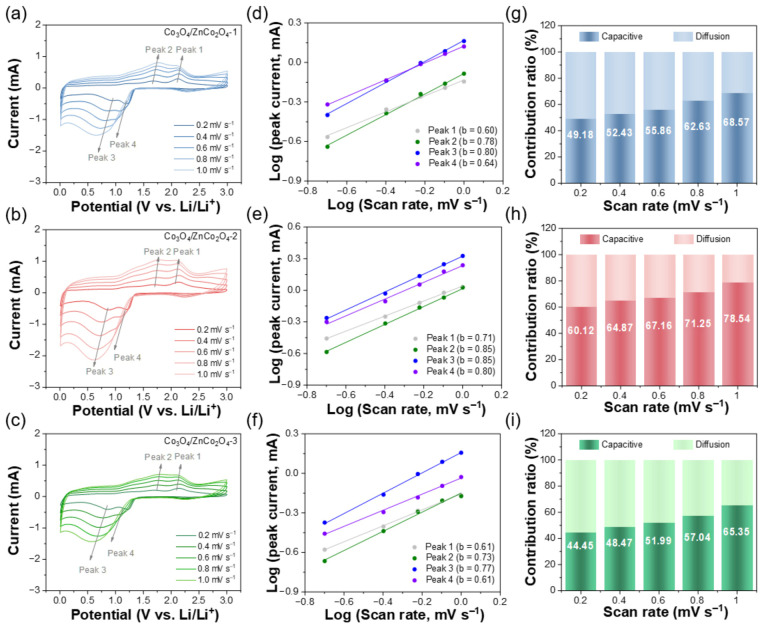
CV curves recorded under various scan rates: (**a**) Co_3_O_4_/ZnCo_2_O_4_-1, (**b**) Co_3_O_4_/ZnCo_2_O_4_-2 and (**c**) Co_3_O_4_/ZnCo_2_O_4_-3. Fitting curves of the b-values: (**d**) Co_3_O_4_/ZnCo_2_O_4_-1, (**e**) Co_3_O_4_/ZnCo_2_O_4_-2 and (**f**) Co_3_O_4_/ZnCo_2_O_4_-3. Contribution rate of capacitance at different scan rates: (**g**) Co_3_O_4_/ZnCo_2_O_4_-1, (**h**) Co_3_O_4_/ZnCo_2_O_4_-2 and (**i**) Co_3_O_4_/ZnCo_2_O_4_-3.

CV tests were performed at different scan rates of 0.01–3.0 V to probe the Li^+^ transport kinetics of the three materials, as shown in Figure 5a–c. All three anodes feature two pairs of distinct reduction and oxidation peaks. The Co_3_O_4_/ZnCo_2_O_4_-2 anode consistently shows the strongest current response. With increasing scan rate, the CV curves of the three anodes remain well-preserved without distortion, indicating low polarization. For the three anodes, the b-value can be derived from the dependence of scan rate (v) and current (i) according to the equation given below [40]:

i = av^b^ (7)

log(i) = b log(v) + log(a)(8)

Generally, when the b-value approaches 0.5, it indicates that diffusion is the dominant driving force for the reaction process; when the b-value approaches 1, it suggests that capacitive effects play a more important role in the reaction [41]. The b-value between 0.5 and 1.0 indicates that the electrochemical reaction is governed by a combination of both mechanisms. The calculated b-values for all three materials fall between 0.5 and 1.0, indicating that their electrochemical reactions are influenced by both diffusion-controlled and capacitive behaviors (Figure 5d–f). For Co_3_O_4_/ZnCo_2_O_4_-2, the b-values for peaks 1, 2, 3, and 4 are 0.71, 0.85, 0.85, and 0.80, respectively, higher than those of Co_3_O_4_/ZnCo_2_O_4_-1 and Co_3_O_4_/ZnCo_2_O_4_-3. This suggests that Co_3_O_4_/ZnCo_2_O_4_-2 exhibits a stronger capacitive contribution, which facilitates charge transfer. At a specific CV scan rate, the percentage of capacitive contribution can be calculated according to the equation given below [42]:

i = k_1_v + k_2_v^1/2^ (9)

Figure 5g–i presents the capacitive contributions of the three anodes determined at different scan rates. With the scan rate rising from 0.2 to 1.0 mV s^−1^, the capacitive contributions progressively increase for all three anodes. At all scan rates, the Co_3_O_4_/ZnCo_2_O_4_-2 anode exhibits a higher capacitive contribution than the other two anodes. At 1.0 mV s^−1^, its capacitive contribution reaches 78.54%, while those of the Co_3_O_4_/ZnCo_2_O_4_-1 and Co_3_O_4_/ZnCo_2_O_4_-3 anodes are only 68.57% and 65.35%, respectively. This demonstrates that interfacial regulation enriches the two-phase interfaces in Co_3_O_4_/ZnCo_2_O_4_-2, promoting interfacial charge redistribution, facilitating charge transfer, and thereby substantially enhancing the capacitive contribution.

**Figure 6:**
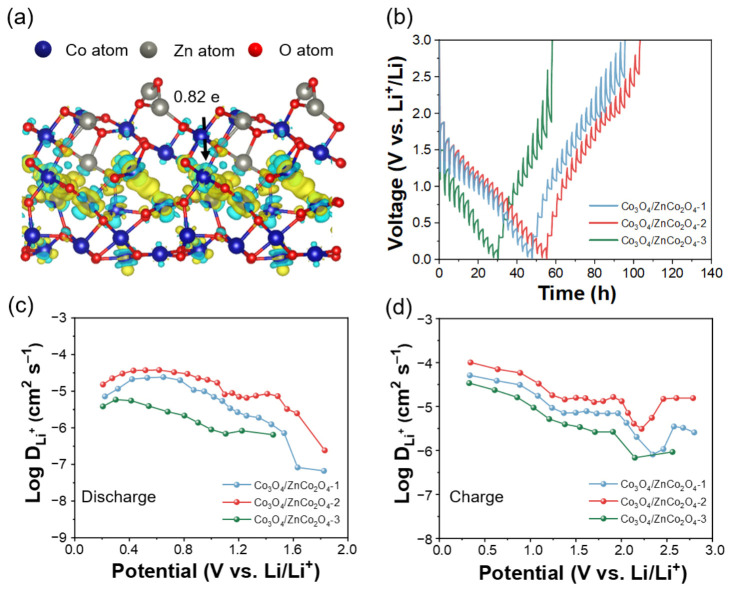
(**a**) The calculated charge density difference of Co_3_O_4_/ZnCo_2_O_4_ heterostructure with corresponding Bader charge. (**b**) GITT profiles. D_Li_^+^ of (**c**) discharge and (**d**) charge.

The impact of heterostructure in facilitating reaction kinetics was further investigated by DFT calculations. The calculated charge density difference of the Co_3_O_4_/ZnCo_2_O_4_ heterointerface is shown in Figure 6a, in which the cyan and yellow electron clouds represent the depletion and accumulation of electrons, respectively. The result indicates that strong charge redistribution behavior occurs at the heterostructure region, yielding a charge transfer of 0.82 e. The observed charge redistribution indicates the presence of a built-in electric field at the interface between Co_3_O_4_ and ZnCo_2_O_4_, which is beneficial for enhancing reaction kinetics and boosting the Li storage capability.

To better understand the diffusion coefficient of lithium-ion (D_Li_^+^) during cycling, the GITT measurements were employed. The materials were subjected to repeated current pulses at 0.1 A g^−1^ for 10 min, with a 30 min relaxation period after each pulse (Figure 6b). At the same current density, Co_3_O_4_/ZnCo_2_O_4_-2 requires a longer duration to complete each cycle, indicating its higher discharge specific capacity. The D_Li_^+^ values during lithiation and delithiation are calculated using Equation (10):

(10) $$D_{{\mathrm{Li}}^{+}}\;=\;\frac{\pi }{\tau }{\left(\frac{n_{m}V_{m}}{A}\right)}^{2}{\left(\frac{{∆E}_{S}}{{∆E}_{\tau }}\right)}^{2}$$

where τ is the current pulse duration, $n_{m}$ represents the molar quantity, $V_{m}$ denotes the molar volume of the electrode, A represents the contact area of the active material with the electrolyte, and ${∆E}_{S}$ and ${∆E}_{\tau }$ denote the steady-state polarization voltage and the total voltage variation over the pulse duration, respectively. The contact area between the active material and the electrolyte is 0.785 cm^2^, and the active material loading and molar volume of three materials used for the calculation are listed in Table S6. Moreover, the calculation assumes semi-infinite diffusion and that the voltage response is proportional to the square root of time during the pulse. Figure 6c,d show the corresponding D_Li_^+^ values of the three anodes upon discharging and charging. During both discharge and charge processes, the Co_3_O_4_/ZnCo_2_O_4_-2 anode exhibits the highest D_Li_^+^. This can be attributed to its abundant two-phase interfaces and oxygen vacancies, which effectively promote electron transport and ion diffusion. However, the other two anodes have lower interface density and lack interfacial active sites, limiting rapid Li^+^ transport.

Figure S4a,b present the EIS data of the three anodes measured before and after 200 cycles at 0.2 A g^−1^. The Nyquist plots show a semicircular shape in the high-frequency region, which is assigned to the charge-transfer resistance (R_ct_). In the low-frequency region, the straight-line segment reflects the Warburg impedance (W_o_) linked to Li^+^ diffusion inside the electrode [43]. Before cycling, the Co_3_O_4_/ZnCo_2_O_4_-2 anode exhibits the smallest R_ct_ and the largest slope in the low-frequency region among the three anodes, indicating its superior Li^+^ transport kinetics. After 200 cycles, the R_ct_ values of all three anodes increase, but Co_3_O_4_/ZnCo_2_O_4_-2 still maintains the lowest R_ct_ with the smallest increase. This suggests that the enhanced electronic reconstruction at the heterointerfaces optimizes the local electronic structure and promotes Li^+^ transfer kinetics.

**Figure 7:**
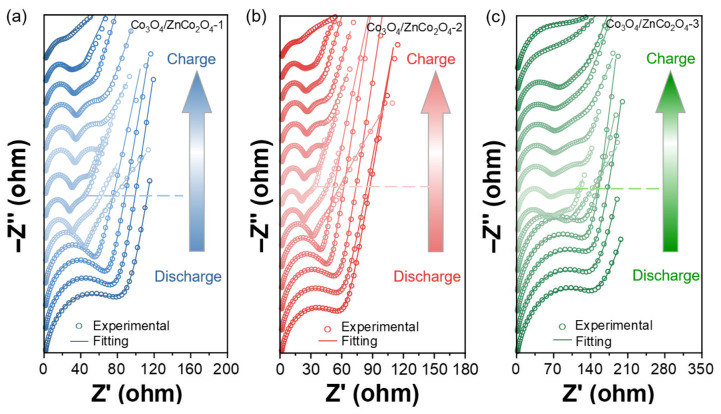
In situ EIS spectra with fitted curves of (**a**) Co_3_O_4_/ZnCo_2_O_4_-1, (**b**) Co_3_O_4_/ZnCo_2_O_4_-2 and (**c**) Co_3_O_4_/ZnCo_2_O_4_-3.

To further investigate the reaction kinetics, in situ EIS tests were conducted on the three anodes during the first charge and discharge processes, with impedance spectra recorded at specific voltage intervals (Figure 7a–c). The dashed line marks the transition between the discharge and charge processes. During the initial discharge, the EIS curve of the Co_3_O_4_/ZnCo_2_O_4_-2 anode exhibits only a semicircle (R_ct_) in the high-frequency region and a linear segment (W_o_) at low frequencies. As the discharge process proceeds, the R_ct_ gradually decreases, while the slope in the low-frequency region remains largely constant. These results indicate that the Co_3_O_4_/ZnCo_2_O_4_-2 anode exhibits fast lithiation reaction kinetics. During the charge process, a new semicircle appears in the high-frequency range, corresponding to the SEI resistance (R_sei_), which remains relatively stable [44,45]. As charging continues, the R_ct_ remains essentially unchanged, the slope in the low-frequency region increases, and the R_sei_ gradually disappears. This suggests that a stable SEI film can rapidly form on the electrode surface, while the heterointerface structure remains stable, facilitating fast Li^+^ transport. Additionally, all EIS spectra have been fitted, and corresponding equivalent circuits are shown in Figure S5. Tables S7–S9 summarize the fitted R_ct_ and R_sei_ results of three anodes. During the charge and discharge processes, the Co_3_O_4_/ZnCo_2_O_4_-2 anode exhibits the lowest R_ct_ values and maintains the smallest R_sei_ among the three anodes. This indicates that the unique two-phase interface of Co_3_O_4_/ZnCo_2_O_4_-2 provides abundant active sites, creating pathways for rapid Li^+^ transport. Additionally, its intact hollow structure and high specific surface area promote adequate electrolyte–electrode contact.

**Figure 8:**
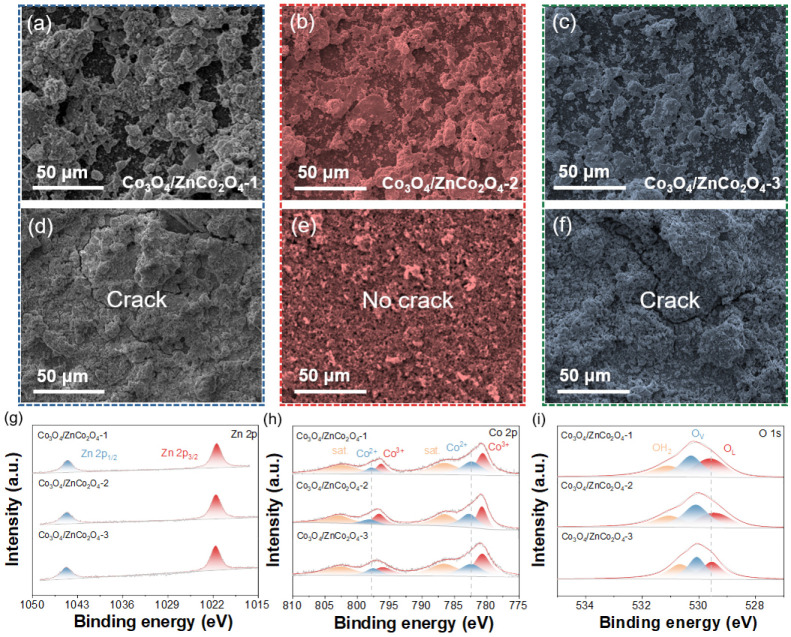
SEM images of anodes before cycling: (**a**) Co_3_O_4_/ZnCo_2_O_4_-1; (**b**) Co_3_O_4_/ZnCo_2_O_4_-2; (**c**) Co_3_O_4_/ZnCo_2_O_4_-3. SEM images of anodes after cycling: (**d**) Co_3_O_4_/ZnCo_2_O_4_-1, (**e**) Co_3_O_4_/ZnCo_2_O_4_-2; (**f**) Co_3_O_4_/ZnCo_2_O_4_-3. High-resolution XPS spectra of (**g**) Zn 2p, (**h**) Co 2p, (**i**) O 1s after 200 cycles.

To evaluate the structural durability of these three electrodes upon cycling, the coin cells were disassembled, and the electrode sheets were characterized by SEM. Comparing the images before and after cycling, the surfaces of all three electrode materials are relatively smooth with no obvious cracks or defects in the pristine state (Figure 8a–c). After 200 cycles over 0.2 A g^−1^, significant differences in surface morphology are observed. The surface of Co_3_O_4_/ZnCo_2_O_4_-2 remains smooth with no visible cracks (Figure 8e). In contrast, the Co_3_O_4_/ZnCo_2_O_4_-1 (Figure 8d) and Co_3_O_4_/ZnCo_2_O_4_-3 (Figure 8f) electrodes exhibit obvious cracks after repeated lithiation and delithiation. The enhanced structural integrity of Co_3_O_4_/ZnCo_2_O_4_-2 is attributed to its intact hollow morphology, which ensures close contact between the conductive agent and the active material, maintains the overall conductive network of the electrode, and thereby preserves the structural integrity of the electrode. In contrast, Co_3_O_4_/ZnCo_2_O_4_-1 and Co_3_O_4_/ZnCo_2_O_4_-3 are less capable of buffering the volume expansion during cycling. Additionally, their limited interfacial interactions lead to stress accumulation inside the electrodes, resulting in structural failure.

The surface chemical states of the three anode materials after 200 cycles at 0.2 A g^−1^ were analyzed. The high-resolution Zn 2p spectra in Figure 8g reveal that Zn remains stably present as Zn^2+^ in all three anodes after cycling, indicating its sustained participation in the alloying reaction, thereby providing additional reversible capacity for the electrode materials. Figure 8h and Figure 8i present the high-resolution Co 2p and O 1s spectra of the three anodes after cycling, respectively. Characteristic peaks of oxygen vacancies are still observable in all samples, providing additional active sites for electrochemical reactions and facilitating Li^+^ insertion/extraction. Moreover, the Co peak remains shifted toward higher binding energy, while the O_L_ peak remains shifted toward lower binding energy, consistent with the results observed before cycling. This indicates that the regulation of the local electronic environment by the heterointerface is effectively preserved. The stable heterointerface provides favorable interfacial conditions for the Co_3_O_4_/ZnCo_2_O_4_-2 to achieve fast Li^+^ transport kinetics.

Table S10 compares the electrochemical performance of recently reported anodes [17,38,39,46,47,48,49,50,51,52,53]. The Co_3_O_4_/ZnCo_2_O_4_-2 anode delivers superior reversible capacity and cycling stability. The outstanding electrochemical properties of Co_3_O_4_/ZnCo_2_O_4_-2 stem from the combined contributions of structure, composition, and heterostructure engineering. In terms of structure, the hollow structure provides ample internal buffer space, effectively mitigating volume expansion, preserving the structural integrity of the electrode throughout cycling. In terms of composition, the introduction of Zn into the Li-Zn alloying reaction not only contributes additional reversible capacity but also lowers the average lithium-insertion potential, thereby improving the energy density. In terms of heterostructure engineering, the optimized phase ratio creates a rich Co_3_O_4_/ZnCo_2_O_4_ heterostructure, which reduces the interfacial charge transfer impedance. The changes in the electronic environment at the phase interface also contribute to improved interfacial charge transport kinetics. Meanwhile, the abundant vacancies introduced modulate the local electronic environment. These aspects collectively contribute to the remarkable lithium storage behavior of the Co_3_O_4_/ZnCo_2_O_4_-2.

**Figure 9:**
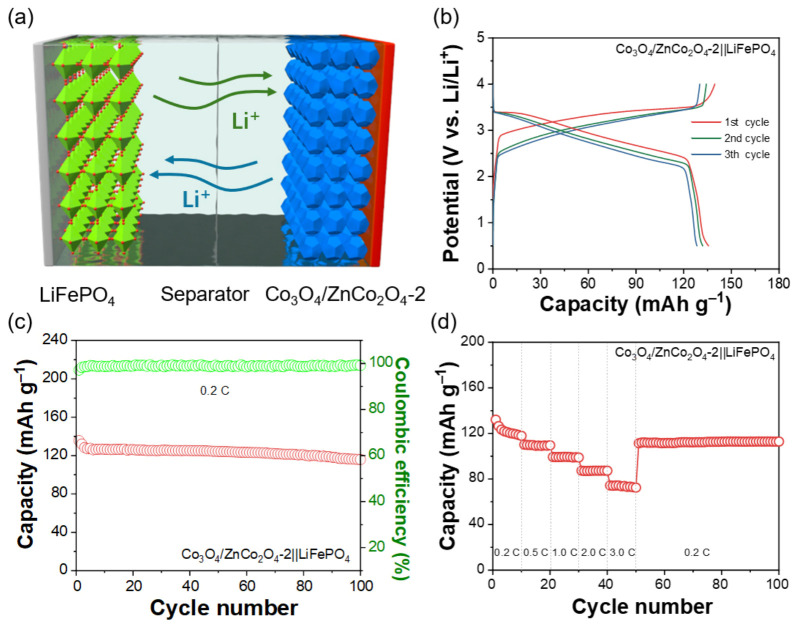
(**a**) Schematic diagram of the Co_3_O_4_/ZnCo_2_O_4_-2||LiFePO_4_ full cell. (**b**) Charge–discharge curves of the Co_3_O_4_/ZnCo_2_O_4_-2||LiFePO_4_ full cell at 0.2 C. (**c**) Cycling performance and (**d**) rate performance of the Co_3_O_4_/ZnCo_2_O_4_-2||LiFePO_4_ full cell.

The Co_3_O_4_/ZnCo_2_O_4_-2 anode was paired with a commercial LiFePO_4_ cathode to assemble a full cell for evaluating its practical application potential (Figure 9a). Figure 9b shows the first three charge–discharge profiles of the full cell at 0.2 C (1 C = 170 mA g^−1^). The initial charge and discharge capacities are 139.6 and 135.6 mAh g^−1^, respectively, with an initial coulombic efficiency of 97.1%. This capacity loss mainly originates from the formation of the cathode electrolyte interphase (CEI) and the decomposition of the electrolyte. As shown in Figure 9c, the Co_3_O_4_/ZnCo_2_O_4_-2||LiFePO_4_ full cell maintains a discharge capacity of 115.9 mAh g^−1^ after 100 cycles at 0.2 C. Furthermore, rate capability tests (Figure 9d) reveal reversible discharge capacities of 132.0, 110.2, 99.3, 87.3, and 74.3 mAh g^−1^ at 0.2, 0.5, 1, 2, and 3 C, respectively. After the current rate is restored to 0.2 C, the capacity returns to 111.4 mAh g^−1^ and remains stable over the subsequent 50 cycles. These results demonstrate the promise of the Co_3_O_4_/ZnCo_2_O_4_-2 material in practical LIBs.

## 4. Conclusions

To summarize, this study developed hollow heterostructured Co_3_O_4_/ZnCo_2_O_4_ anodes with tunable two-phase ratios by adjusting the Zn/Co molar ratio. The optimal Co_3_O_4_/ZnCo_2_O_4_-2 material integrates an intact hollow dodecahedral morphology with abundant two-phase heterointerfaces, collectively buffering volume expansion, promoting interfacial charge transfer, and enhancing structural stability. Furthermore, the oxygen vacancies induced by Zn^2+^ substitution and interfacial charge redistribution significantly improve reaction kinetics and Li^+^ diffusion. DFT calculations further confirm that charge redistribution occurs at the Co_3_O_4_/ZnCo_2_O_4_ heterointerface, facilitating interfacial charge transfer. Consequently, the Co_3_O_4_/ZnCo_2_O_4_-2 anode delivers 863.4 mAh g^−1^ after 200 cycles at 0.2 A g^−1^, and exhibits excellent rate capability. The Co_3_O_4_/ZnCo_2_O_4_-2||LiFePO_4_ full cell demonstrates promising application potential, maintaining a discharge capacity of 115.9 mAh g^−1^ after 100 cycles at 0.2 C. This work demonstrates that the cooperative effect of interfacial charge redistribution and oxygen vacancies, enabled by hollow structure and heterointerface design, is the key factor promoting Li^+^ transport. This mechanistic understanding provides a new perspective on how interfacial engineering enhances the electrochemical performance of bimetallic oxide anodes.

## Data Availability

The original contributions presented in this study are included in the article/Supplementary Materials. Further inquiries can be directed to the corresponding author.

## Supplementary Materials

The following supporting information can be downloaded at: https://www.mdpi.com/article/10.3390/ma19143148/s1, Supporting S1. Experimental details. Figure S1. SEM image of Zn/Co-ZIF. Figure S2. The GCD curves of (a) Co_3_O_4_/ZnCo_2_O_4_-1 and (b) Co_3_O_4_/ZnCo_2_O_4_-3 at 0.2 A g^–1^. Figure S3. Data stability tests. (a) Discharge capacities of Co_3_O_4_/ZnCo_2_O_4_-2 from the same batch. (b) Standard deviations of the data in (a) at different cycle numbers. Figure S4. EIS spectra of three anodes: (a) Before and (b) after cycling for 200 cycles at 0.2 A g^–1^. Figure S5. Equivalent circuits of in situ EIS spectra of Figure 7. (a) The R_ct_ fitting. (b) The R_ct_ and R_sei_ fitting. Table S1. The EDS elemental analysis of the three Co_3_O_4_/ZnCo_2_O_4_ samples. Table S2. The ICP results of the three Co_3_O_4_/ZnCo_2_O_4_ samples. Table S3. Quantitative XPS analysis of Co^2+^/Co^3+^ and O_V_/O_L_ ratios. Table S4. Areal capacities of the three anodes at 0.2 A g^–1^ after 200 cycles. Table S5. Data stability of three batches of batteries. Table S6. Parameters used for the GITT calculation. Table S7. Fitted EIS results of Co_3_O_4_/ZnCo_2_O_4_-1. Table S8. Fitted EIS results of Co_3_O_4_/ZnCo_2_O_4_-2. Table S9. Fitted EIS results of Co_3_O_4_/ZnCo_2_O_4_-3. Table S10. The comparison of electrochemical performances with previous works.
