# Supplementary material for: Boosting Reaction Kinetics in Co3O4/ZnCo2O4 Frameworks with Heterostructures for High-Performance Lithium-Ion Batteries
Source: Materials (Basel). 2026 Jul 22;19(14):3148. doi: 10.3390/ma19143148 (PMC13414429; doi:10.3390/ma19143148)
Supplement: Supplementary file 1 [file materials-19-03148-s001.zip › materials-4404729-supplementary.pdf]

Supporting information

Boosting Reaction Kinetics in $Co_3O_4/ZnCo_2O_4$ Frameworks with Heterostructures for High-Performance Lithium-Ion Batteries

Qibei Tu and Zhifeng Wang *

"The Belt and Road Initiative" Advanced Materials International Joint Research Center of Hebei Province, School of Materials Science and Engineering, Hebei University of Technology, Tianjin 300401, China

Supporting S1 Experimental details

Material characterization

The morphology and structure were characterized using scanning electron microscopy (SEM, Quanta 450 FEG, FEI, Hillsboro, OR, USA) and transmission electron microscopy (TEM, JEM 2100F, JEOL, Tokyo, Japan). Crystallographic data were analyzed using a D8 Advance X-ray diffractometer (XRD, Karlsruhe, Germany) from Bruker company. Raman spectroscopy was conducted using the LabRAM HR Evolution system (HORIBA, Palaiseau, France). The vacancy analysis was performed on a Bruker EMXplus spectrometer (Karlsruhe, Germany). N_2 adsorption-desorption isotherms were carried out on the ASAP 2460 system of Micromeritics company (Norcross, GA, USA). X-ray photoelectron spectroscopy (XPS, Nexsa G2, Waltham, MA, USA) was employed to analyze the composition and chemical states of the samples.

Electrochemical tests

Coin cells were assembled to assess the electrochemical behavior of the as-prepared samples. A mixture containing the active material, carboxymethyl cellulose (CMC), and Super P at a weight ratio of 7:2:1 was prepared in deionized water. The obtained slurry was spread onto copper foil and dried at 60 °C. A lithium metal foil served as the counter electrode, with a Celgard 2325 polypropylene membrane (thickness: 25 μm) as the separator. The electrolyte consisted of 1 M $LiPF_6$ dissolved in an ethylene carbonate/dimethyl carbonate mixture (EC:DEC = 1:1 by volume). The active material loading was approximately 0.9–1.1 mg cm^{-2}, and the electrode thickness was about 14 μm. The amount of electrolyte used in each coin cell was 50 μL. Charge and discharge measurements were carried out on a Neware CT-4000 battery testing system (Shenzhen Neware Electronics Co., Ltd., Shenzhen, China) within a voltage window of 0.01–3.0 V. Cyclic voltammetry (CV) and electrochemical impedance spectroscopy (EIS) were performed using a CHI 660E electrochemical workstation (Shanghai Chenhua Instrument Co., Ltd., Shanghai, China). The DH 7000D workstation (Jiangsu Donghua Analytical Instrument Co., Ltd., Taizhou, China) was used for in situ EIS tests.

The prepared anode was assembled into full cells with commercial $LiFePO_4$ (CAS: 15365–14–7) cathodes for electrochemical performance testing. The cathodes were prepared as follows: LFP powder, Super P, and polyvinylidene fluoride (PVDF) were blended at an 8:1:1 weight ratio. N-methylpyrrolidone (NMP) was then introduced as the solvent in an appropriate amount. The mixture was fully ground until a homogeneous slurry formed, then coated onto a carbon-coated aluminum foil. The mass loading of the $LiFePO_4$ cathode was approximately 5.8–6.2 mg cm^{-2} with an electrode thickness of about 45 μm, and the N/P ratio was 1.05–1.1. The amount of electrolyte used in each coin cell was 50 μL. Prior to assembling the full cell, the anode material was prelithiated by galvanostatic discharge to 0.05 V, and then was paired with the $LiFePO_4$ cathode for full-cell testing. The capacity of the full cell was calculated based on the mass of the $LiFePO_4$ cathode, using a theoretical specific capacity of 170 mAh g^{-1} for normalization. Galvanostatic charge/discharge tests of the full cells were conducted at 0.5–4.0 V.

Theoretical calculation

All the first-principle calculations were performed based on the spin-polarized density functional theory (DFT) using the Vienna ab initio simulation package (VASP) [21,22]. The electron exchange correlation was described by the gradient-corrected Perdew-Burke-Ernzerhof (GGA-PBE) functional [23]. Ion-electron interaction was described by the projector augmented-wave (PAW) method [24]. The cutoff energy was set to 500 eV, and the total energy and force convergence for geometric optimization was set to $2*10^{-5}$ eV and 0.05 eV/Å, respectively. The vacuum layer was set to 15 Å in the z direction and a semi-empirical correction of DFT-D3 was adopted to account for the interaction of van der Waals forces [25].

The charge density difference was calculated by: $\Delta\varrho = \varrho ZnCo_2O_4/Co_3O_4 - \varrho Co_3O_4 - \varrho ZnCo_2O_4$, where $\varrho ZnCo_2O_4/Co_3O_4$, ϱCo_3O_4 and $\varrho ZnCo_2O_4$ are the charge densities of $ZnCo_2O_4/Co_3O_4$ heterostructure, the Co_3O_4 and $ZnCo_2O_4$ monolayers, respectively. The isosurface value is set as 0.004 e $Å^{-3}$.

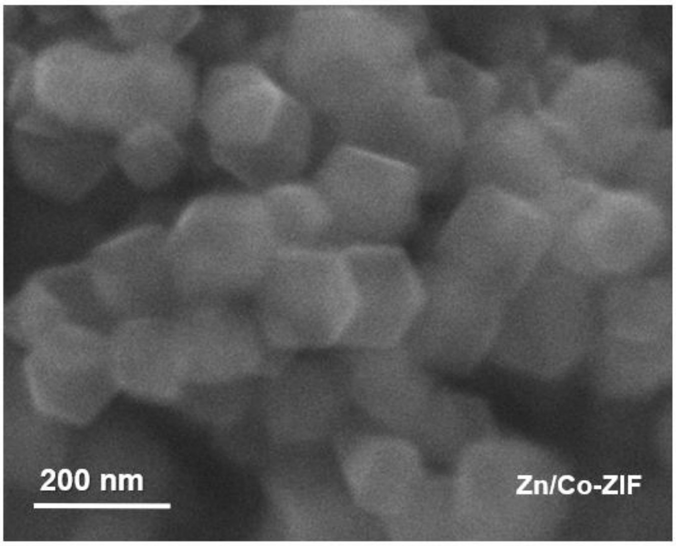

Figure S1. SEM image of Zn/Co-ZIF.

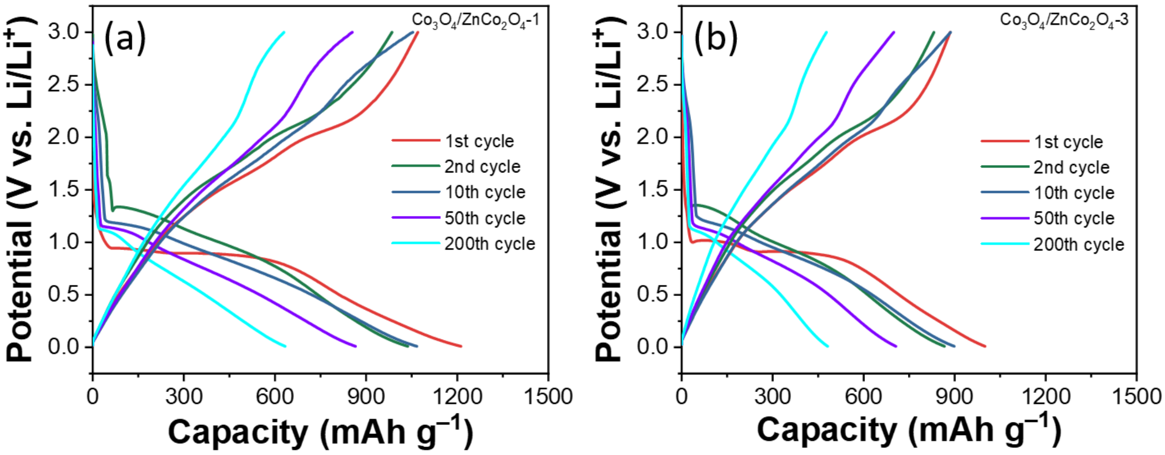

Figure S2. The GCD curves of (**a**) $Co_3O_4/ZnCo_2O_4$-1 and (**b**) $Co_3O_4/ZnCo_2O_4$-3 at 0.2 A g^{-1}.

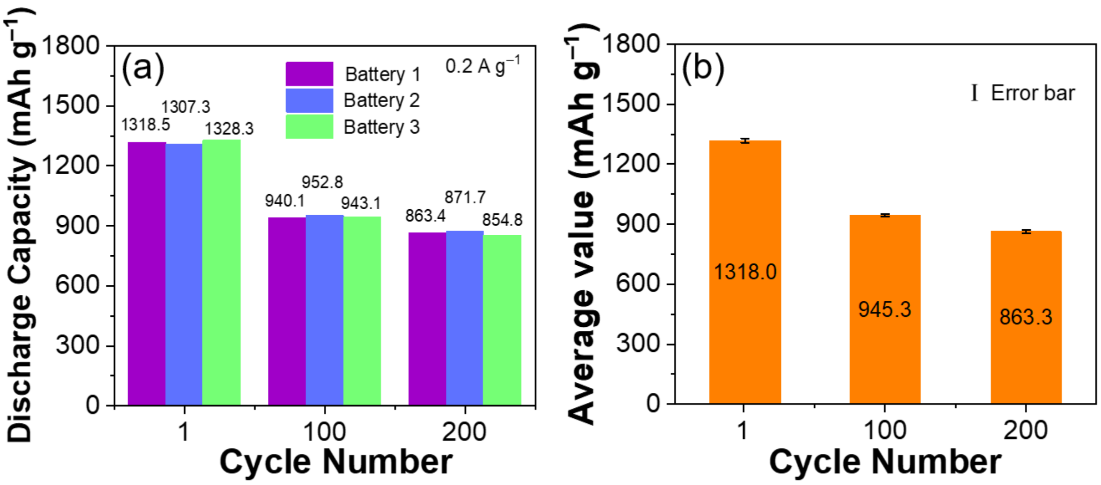

Figure S3. Data stability tests. (**a**) Discharge capacities of $Co_3O_4/ZnCo_2O_4$-2 from the same batch. (**b**) Standard deviations of the data in (a) at different cycle numbers.

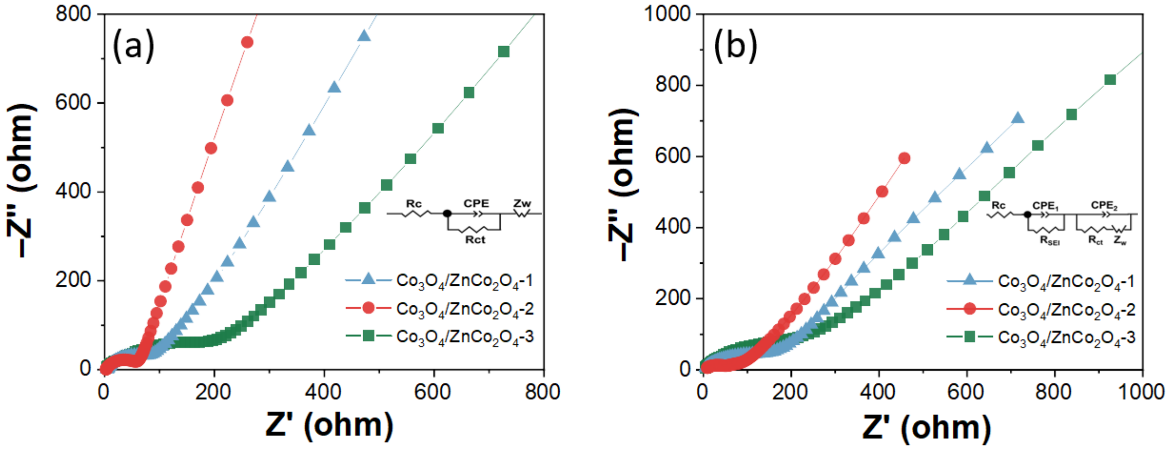

Figure S4. EIS spectra of three anodes: (**a**) Before and (**b**) after cycling for 200 cycles at 0.2 A g^{-1}.

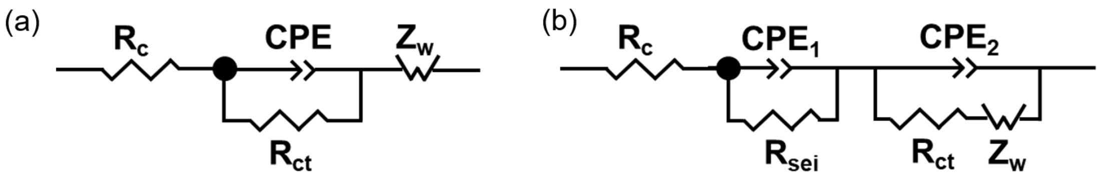

Figure S5. Equivalent circuits of in situ EIS spectra of Figure 7. **(a)** The R_{ct} fitting. (**b**) The R_{ct} and R_{sei} fitting.

Table S1 The EDS elemental analysis of the three $Co_3O_4/ZnCo_2O_4$ samples.

**Materials**	**Zn Atomic %**	**Co Atomic %**	**O Atomic %**
$Co_3O_4/ZnCo_2O_4$-1	12.9	32.8	54.3
$Co_3O_4/ZnCo_2O_4$-2	10.6	41.0	48.4
$Co_3O_4/ZnCo_2O_4$-3	9.8	43.3	46.9

Table S2 The ICP results of the three $Co_3O_4/ZnCo_2O_4$ samples.

**Materials**	**Zn wt %**	**Co wt %**
$Co_3O_4/ZnCo_2O_4$-1	18.9	43.2
$Co_3O_4/ZnCo_2O_4$-2	15.2	52.1
$Co_3O_4/ZnCo_2O_4$-3	13.8	53.9

Table S3 Quantitative XPS analysis of Co^{2+}/Co^{3+} and O_V/O_L ratios.

**Materials**	**Co^{2+}/Co^{3+}**		**O_V/O_L**
	Co $2p_{1/2}$	Co $2p_{3/2}$	
$Co_3O_4/ZnCo_2O_4$-1	1.09	1.00	0.93
$Co_3O_4/ZnCo_2O_4$-2	1.10	1.05	0.98
$Co_3O_4/ZnCo_2O_4$-3	1.15	1.09	0.95

Table S4 Areal capacities of the three anodes at 0.2 A g^{-1} after 200 cycles.

**Materials**	**Areal capacity (mAh cm^{-2})**
$Co_3O_4/ZnCo_2O_4$-1	0.60
$Co_3O_4/ZnCo_2O_4$-2	0.87
$Co_3O_4/ZnCo_2O_4$-3	0.47

Table S5 Data stability of three batches of batteries.

Current density (A g^{-1})	Cycle number	Batch 1					Batch 2					Batch 3					All batches	
		Battery 1	Battery 2	Battery 3	AV	SD	Battery 4	Battery 5	Battery 6	AV	SD	Battery 7	Battery 8	Battery 9	AV	SD	AV	SD
0.2	1	1318.5	1307.3	1328.3	1318.0	10.5	1311.6	1324.2	1309.4	1315.1	8.0	1321.7	1315.4	1322.3	1319.8	3.8	1317.6	7.2
	100	940.1	952.8	943.1	945.3	6.6	934.9	947.8	936.1	939.6	7.1	933.8	945.5	931.7	937.0	7.4	940.6	7.2
	200	863.4	871.7	854.8	863.3	8.5	869.8	876.0	860.1	868.6	8.0	856.7	868.9	861.2	862.3	6.2	864.7	7.1
1.0	1	1206.0	1203.5	1198.7	1202.7	3.7	1211.4	1207.9	1196.1	1205.1	8.0	1200.9	1193.1	1201.5	1198.5	4.7	1202.1	5.8
	500	693.8	699.7	701.9	698.5	4.2	703.9	688.4	690.1	694.1	8.5	682.4	695.2	710.6	696.1	14.1	696.2	8.7
	1000	582.4	594.3	585.0	587.2	6.3	580.7	579.1	588.3	582.7	4.9	589.0	577.3	582.1	582.8	5.9	584.2	5.4
Note:	AV: average value; SD: standard deviation																	

Table S6 Parameters used for the GITT calculation.

Materials	Active material loading (mg cm^{-2})	Molar volume (cm^3 mol^{-1})
$Co_3O_4/ZnCo_2O_4$-1	0.95	39.91
$Co_3O_4/ZnCo_2O_4$-2	1.01	39.87
$Co_3O_4/ZnCo_2O_4$-3	0.97	39.86

Table S7 Fitted EIS results of $Co_3O_4/ZnCo_2O_4$-1.

$Co_3O_4/ZnCo_2O_4$-1	R_{ct} (Ω)	R_{sei} (Ω)	Fitting error (%)
Discharge	65.75	–	1.2872
	61.5	–	1.1007
	59.01	–	1.2235
	51.34	–	1.3343
	38.28	43.04	1.0721
	26.22	29.13	0.9927
Charge	17.17	26.52	0.4589
	17.59	29.39	0.3637
	23.14	32.74	0.3768
	24.89	34.21	1.022
	31.61	34.61	1.1006
	20.12	32.67	0.808
	33.34	–	1.6722
	37.21	–	1.0747

Table S8 Fitted EIS results of $Co_3O_4/ZnCo_2O_4$-2.

$Co_3O_4/ZnCo_2O_4$-2	R_{ct} (Ω)	R_{sei} (Ω)	Fitting error (%)
Discharge	46.94	–	1.1152
	45.6	–	1.0921
	45.24	–	1.0285
	41.59	–	1.2174
	32.31	33.91	0.9098
	26.34	28.48	1.1548
	19.96	21.77	0.4179
Charge	6.298	19.81	0.6614
	13.15	22.22	0.7669
	16.91	24.22	0.5002
	17.77	25.26	0.7624
	17.99	25.96	0.8237
	19.16	24.73	2.6212
	19.27	23.98	1.0808
	26.03	–	2.6022

Table S9 Fitted EIS results of $Co_3O_4/ZnCo_2O_4$-3.

$Co_3O_4/ZnCo_2O_4$-3	R_{ct} (Ω)	R_{sei} (Ω)	Fitting error (%)
Discharge	113.7	–	1.4175
	113.2	–	1.9216
	116.4	–	1.4152
	113.2	–	1.5285
	103.3	–	1.3682
	82.38	–	1.534
	65.28	50.96	1.0342
Charge	67.35	53.84	1.6088
	58.39	59.51	1.7576
	63.26	60.18	1.6504
	57.72	61.33	0.6226
	56.62	56.62	1.3079
	61.18	–	0.9019
	73.59	–	0.9662
	84.03	–	1.0479

Table S10 The comparison of electrochemical performances with previous works.

Anode materials	Mass loading (mg cm^{-2})	ICE	Current density (mA g^{-1})	Cycle number	Specific capacity (mAh g^{-1})	References
$CoSe_2$@NC	1.0–1.5	75.7 %	100	30	699.0	[46]
$ZnCo_2O_4$	–	–	100	200	825.0	[38]
$MoS_2/ZnCo_2O_4$	–	73.7 %	100	200	736.0	[39]
$ZnCo_2O_4$ (BDC-ZCO)	1.00	65.5 %	100	200	695.1	[47]
SnO_2-Co_3O_4	–	76.5 % (0.1 A g^{-1})	200	100	767.5	[48]
$ZnCo_2O_4$/GA	0.65	64.2 % (0.1 A g^{-1})	1000	400	591.0	[49]
Sn/SnO_x@NC	1.00	66.4 %	1000	500	487.3	[50]
NiO-Co_3O_4@rGO	–	60.5 % (0.1 A g^{-1})	1000	500	434.1	[51]
NiO/Co_3O_4	–	68.3 % (0.1 A g^{-1})	1000	600	668.6	[17]
$FeSe_2$-Fe_2O_3@GA	1.20	60.0 % (0.1 A g^{-1})	1000	600	492.7	[52]
Co_3O_4-CTS/MXene	–	62.6 %	1000	800	332.9	[53]
$Co_3O_4/ZnCo_2O_4$-2	0.9–1.1	89.9 %	200	200	863.4	This work
		88.3 %	1000	600	675.8	
			1000	1000	582.4	
